# TGFBR3 regulates the expression of FATP2-ACSL1 axis related proteins and accompanies changes in the migration and invasion ability of lung cancer cells

**DOI:** 10.3389/fonc.2026.1899185

**Published:** 2026-08-12

**Authors:** Tian Luo, Yuanyuan Wu, Nuoya Ma, Junyu He, Wenling Zhang, Guoying Zou

**Affiliations:** 1 College of Clinical Medicine, Hunan University of Chinese Medicine, Changsha, Hunan, China; 2 Department of Clinical Laboratory, The Second People’s Hospital of Hunan Province (Brain Hospital of Hunan Province), Changsha, Hunan, China; 3 Laboratory Department, Changsha County Hospital of Traditional Chinese Medicine, Changsha, Hunan, China; 4 Department of Medical Laboratory Science, The Third Xiangya Hospital, Central South University, Changsha, Hunan, China; 5 Department of Medical Laboratory Science, Xiangya School of Medicine, Central South University, Changsha, Hunan, China

**Keywords:** FATP2, fatty acid metabolism, invasion and migration, lung cancer, TGFBR3

## Abstract

**Background:**

Abnormal lipid metabolism promotes lung cancer progression. This study explores how TGFBR3 regulates fatty-acid-metabolism-related protein expression via the FATP2-ACSL1 axis to suppress lung cancer invasion and metastasis, and preliminarily assesses the potential clinical value of TGFBR3 and FATP2.

**Methods:**

Serum samples were collected from 30 healthy individuals and 170 newly diagnosed lung cancer patients for lipid profiling. Network pharmacology, Mendelian randomization, molecular docking, co-immunoprecipitation (Co-IP) and GST-Pull down assay were used to validate the TGFBR3-FATP2 interaction. Stable cell lines were established to assess function and mechanism via CCK-8, wound healing, Transwell, and Western blotting. Serum levels of soluble TGFBR3 (sTGFBR3) and FATP2 were measured by ELISA.

**Results:**

Serum CHOL, HDL-C, and LDL-C were significantly decreased in lung cancer patients (*P* < 0.05). Mendelian randomization indicated that elevated HDL-C reduces lung cancer risk (OR = 0.5607). Molecular docking showed a binding energy of −12.5 kcal/mol, Co-IP and GST-Pull down assay confirmed the interaction. TGFBR3 overexpression inhibited migration and invasion, while knockdown had the opposite effect (*P* < 0.05). TGFBR3 regulated FATP2, ACC1, ACSL1, and SCD1 expression but not CD36. Lipofermata reversed the protein changes induced by TGFBR3 knockdown. ELISA results showed that serum sTGFBR3 and FATP2 levels were significantly higher in lung cancer patients than in healthy and pneumonia controls (*P* < 0.05), and sTGFBR3 levels were higher in LUSC and SCLC than in LUAD (*P* < 0.05). The AUCs were 0.931 for sTGFBR3 and 0.998 for FATP2, showing higher diagnostic performance compared with CEA and other traditional markers in this cohort. Furthermore, sTGFBR3 levels decreased after carboplatin dose reduction or discontinuation and increased after treatment resumption; *in vitro* experiments confirmed that carboplatin upregulated TGFBR3 expression and promoted the release of its soluble form (*P* < 0.05). although this effect may be partly attributed to nonspecific cell injury.

**Conclusions:**

TGFBR3 interacts with FATP2 and is associated with the regulation of migration and invasion in lung cancer cells, potentially involving the FATP2-ACSL1 axis. Additionally, serum levels of sTGFBR3 and FATP2 were identified as potential candidate biomarkers for lung cancer, warranting further validation in independent cohorts migration.

## 1 Introduction

According to the Global Cancer Statistics 2022, lung cancer ranks first in both incidence and mortality rates (12.4% and 18.7%, respectively) (1). Approximately 75% of lung cancer patients are diagnosed at an advanced stage or with distant migration, and migration remains the leading cause of death (2). Among patients with non-small cell lung cancer (NSCLC), approximately 23% to 36% develop brain migration (3). Therefore, investigating the mechanisms of invasion and metastasis of lung cancer and identifying effective biomarkers has important clinical significance.

Our preliminary clinical observations revealed that newly diagnosed lung cancer patients exhibit dysregulated lipid metabolism, manifested as significant decreases in total cholesterol, high-density lipoprotein cholesterol, and low-density lipoprotein cholesterol (*P* < 0.05). Multiple studies have confirmed that aberrant activation of lipid metabolism promotes tumor proliferation and metastasis (4–6). Bioinformatics analysis combined with Mendelian randomization showed that a common target involved the TGF - β family and fatty acid synthase, and HDL-C was negatively correlated with lung cancer risk (*P* < 0.05),and the TGF-β signaling pathway may regulate the expression of fatty acid metabolism-related proteins (7, 8); however, the underlying mechanism remains unclear.

Our research group has long been investigating the invasion and metastasis of lung cancer. Previous findings showed that transforming growth factor beta receptor III (TGFBR3), the co-receptor of the TGF-β family member inhibin B, is downregulated in lung cancer cells, and its downregulation is associated with clinical stage and lymph node metastasis (9). As a co-receptor of the TGF-β superfamily, TGFBR3 functions as a tumor suppressor in most tumors (10). In lung cancer, its mRNA and protein levels are significantly reduced (11, 12), particularly in lung adenocarcinoma and lung squamous cell carcinoma, where its decreased expression correlates with patient prognosis (9, 13). Mechanistically, TGFBR3 inhibits epithelial-mesenchymal transition (EMT) to prevent tumor invasion and metastasis (14). Another study indicated that increased soluble TGFBR3 (sTGFBR3) induces EMT; however, this EMT is associated with inhibited invasion and metastasis but accelerated tumor growth, demonstrating the bifunctional role of TGFBR3 (15). In addition to its well-established roles in regulating EMT and tumor progression, TGF-β signaling has also been implicated in metabolic reprogramming. For instance, a recent study by Liu et al. demonstrated that IMUP promotes fatty acid metabolism and lung cancer progression through activating the TGF-β/SMAD3 signaling pathway (16), further supporting the crosstalk between TGF-β signaling and fatty acid metabolic reprogramming in lung cancer.

Fatty acid transport protein 2 (FATP2) is a key protein responsible for transmembrane fatty acid transport, possessing dual functions as a transporter and a long-chain acyl-CoA synthetase (17, 18). Studies have shown that FATP2 is overexpressed in NSCLC and correlates with poor prognosis, promoting tumor progression through ACSL1-mediated regulation of lipid metabolism (19); however, its upstream regulatory mechanism remains unclear. A recent study by Chen et al. demonstrated that ACSL5, an enzyme involved in long-chain fatty acid metabolism, functions as an immune-dependent tumor suppressor by enhancing MHC-I-mediated antigen presentation and sensitizing tumors to PD-1 blockade therapy (20). Furthermore, beyond its role in cancer, fatty acid oxidation has also been implicated in immune regulation in non-malignant contexts. For instance, a recent study by Wu et al (21). demonstrated that mitochondrial long-chain fatty acid β-oxidation (mtLCFAO) in type 2 alveolar epithelial (AT2) cells plays a critical role in mitigating neutrophilic inflammation during acute lung injury, with impaired mtLCFAO functioning as an anti-inflammatory response that restricts alveolar inflammation by hindering CXCL2 production. Collectively, these studies highlight the diverse and context-dependent roles of fatty acid metabolic pathways in both tumor immunity and inflammatory lung diseases, further supporting the rationale for investigating the interplay between fatty acid metabolism and immune regulation in the lung microenvironment.

In summary, given the tumor suppressor role of TGFBR3 in lung cancer, we hypothesized that TGFBR3 may interact with lipid metabolism-related proteins. As an exploratory investigation, network pharmacology analysis and Mendelian randomization analysis were conducted to identify potential lipid-related traits associated with lung cancer risk. Through molecular docking and co-immunoprecipitation experiments, FATP2 was preliminarily identified as a putative interacting partner of TGFBR3. Further cellular functional experiments and mechanistic validation were performed to explore how these two proteins regulate fatty-acid-metabolism-related protein expression and subsequently affect lung cancer cell invasion and migration. Additionally, serum levels of sTGFBR3 and FATP2 were measured in healthy controls, pneumonia patients, and newly diagnosed lung cancer patients as an exploratory assessment of their possible clinical relevance.

## 2 Methods

### 2.1 Fully automatic biochemical analyzer

Serum levels of total cholesterol (CHOL), high-density lipoprotein cholesterol (HDL-C), low-density lipoprotein cholesterol (LDL-C), triglycerides (TG), apolipoprotein A1 (ApoA1), apolipoprotein B (ApoB), and lipoprotein(a) [Lp(a)] were measured using a Siemens Advia 2400 automated biochemical analyzer. Calibration and quality control were performed prior to analysis. Serum samples were collected by centrifugation and quantified using colorimetric methods according to the manufacturer’s instructions. All batch measurements met the required quality control criteria.

### 2.2 Network pharmacology analysis and Mendelian randomization

Using “lipid metabolism disorder” and “lung cancer” as keywords, search for relevant targets in GeneCards, CTD, and OMIM databases, and take the intersection to obtain common targets. Construct a protein-protein interaction (PPI) network using the STRING database, with species limited to humans and a confidence threshold set at 0.400. Using CytoScape 3.9.1 software, core targets were selected based on node degree values, betweenness centrality, and compactness centrality all being higher than the median. Subsequently, KEGG pathway enrichment analysis was performed on the core targets using the DAVID database (*P* < 0.05, FDR<0.05) to screen for tumor related signaling pathways (**Table 1**).

**Table 1:** Database.

Network analysis platform	URL	Filter criteria
UniProt	https://www.uniprot.org/	Homo sapiens
GeneCards	https://www.genecards.org	Relevance score≥ median
CTD	http://ctdbase.org/	Relevance score≥ median
OMIM	https://www.omim.org/	Relevance score≥ median
Venn online tool	http://bioinformatics.psb.ugent.be/	
STRING	https://cn.string-db.org/	Homo sapiens and score ≥0.4
DAVID	https://davidbioinformatics.nih.gov/	
Cytoscape 3.9.1	https://cytoscape.org/	Degree≥53.42, Betweenness Centrality≥116.64, Closeness Centrality≥0.00364
Weishengxin online tool	https://www.bioinformatics.com.cn/	Degree

Using a two sample Mendelian randomization method and publicly available GWAS summary data, the causal relationship between lipid metabolism related traits (high-density lipoprotein cholesterol, low-density lipoprotein cholesterol, triglycerides, total cholesterol, apolipoprotein A1 and apolipoprotein B) and lung cancer was analyzed. Single nucleotide polymorphisms (SNPs) were selected as instrumental variables at the genome-wide significance level (P < 5 × 10^-8^). To ensure the independence of the selected genetic variants, linkage disequilibrium was removed based on the 1000 Genomes Project European reference panel with an R² threshold of 0.001 and a clumping window of 10,000 kb. Palindromic SNPs with intermediate allele frequencies were also excluded to avoid ambiguity in allele orientation. The main analysis method is the inverse variance weighting method, supplemented by the weighted median method and MR Egger regression. Heterogeneity and pleiotropy were evaluated using Cochran’s Q test, MR Egger intercept test, leave one method, and MR-PRESSO method. Statistical analysis was conducted using R software, with *P* < 0.05 as the statistical significance standard. This study conducted preliminary MR analysis on six lipid traits. Considering that the MR analysis results of the remaining five lipid traits were all negative, this article only reports the association results between HDL-C and lung squamous cell carcinoma.

### 2.3 Molecular docking

The structure of the protein comes from the RCSB PDB database(https://www.pdbus.org/)The PDB format in (22). The structure of small molecule ligands was obtained from public compound databases such as PubChem. Molecular docking between the two proteins (TGFBR3 and FATP2) was performed using ZDOCK 3.0.2 (23) with default parameters for global rigid-body docking. The initial docking poses were subsequently subjected to energy minimization using the AMBER18 software package with the ff14SB force field. The binding affinity of the minimized protein-protein complex was evaluated using the PRODIGY web server (https://wenmr.science.uu.nl/prodigy/) (25). The complex conformation with the optimal binding energy was selected for visualization and analysis of interface residues using PyMOL 2.5.3 (24).

For protein-small molecule docking, the 3D structures of ligands were prepared, and protein structures were processed (including removal of water molecules and format conversion) using Chem3D and PyMOL. Docking calculations were carried out using AutoDock Tools, which involved the addition of polar hydrogens, assignment of Gasteiger charges, and setting of grid boxes. The resulting docking conformations were visualized in PyMOL for three-dimensional structural representation.

The three-dimensional structure of TGFBR3 was retrieved from the Protein Data Bank (PDB) under accession code 7LBG. The structure of FATP2 was obtained from the AlphaFold Protein Structure Database (entry AF-O14975-FATP2). Chemical compounds used in this study were sourced from the PubChem database with the following Compound IDs (CIDs) or Substance IDs (SIDs): cholesterol (CID: 5997), DG (CID: 117791079), linoleic acid (CID: 5280450), Lysophosphatidylcholine (CID: 5311264), oleic acid (CID: 445639), and LPI (SID: 160645163).

### 2.4 Cell culture and transfection

The human lung adenocarcinoma cell line A549 and the human lung squamous cell carcinoma lymph node metastasis cell line NCI-H292 were both purchased from the laboratory of the Advanced Research Center (Modern Analysis and Testing Center) of Central South University. A549 is a well-characterized alveolar adenocarcinoma cell line widely used in lung cancer research, while NCI-H292 is derived from a pulmonary metastasis of mucoepidermoid carcinoma, providing a distinct histological background for comparative validation. All experiments were conducted with mycoplasma-negative cells. Cultivate in RPMI-1640 medium containing 10% fetal bovine serum and 1% penicillin streptomycin at 37 °C in a 5% CO2 incubator. The E. coli strains carrying TGFBR3 overexpression lentivirus (LV-TGFBR3101491-2) and TGFBR3 interference lentivirus (LV-TGFBR3-RNAi, 110414-1) and their corresponding negative controls were purchased from Shanghai Jikai Gene (Shanghai, China). After transducing the above-mentioned lentivirus into NCI-H292 and A549 cells, stable transfected cell lines were screened using Puromycin (Shanghai Biyuntian). The transduction efficiency was evaluated by observing the expression of green fluorescent protein (GFP) under a fluorescence microscope, and the expression level of TGFBR3 was verified by Western blot.

OE-TGFBR3: A plasmid used for overexpression was con structed by GeneChem, in which the TGFBR3 (NM_003243.5) gene was cloned into the GV492 vector.

OE-TGFBR3-NC: CON335 from GeneChem.

sh-TGFBR3: CCAAGCATGAAGGAACCAAAT;

sh-TGFBR3-NC: TTCTCCGAACGTGTCACGT;

### 2.5 Co-IP experiment

The cell lysate was sonicated and centrifuged to obtain protein supernatant. The target antibody was added and incubated overnight at 4 °C. Wash Protein A/G agarose beads with IP lysis buffer 4 times, then slowly shake and incubate with the above antibody protein mixture at 4 °C for 2 hours. Centrifuge and discard the supernatant, wash the agarose beads 4 times with IP lysis buffer, and the resulting precipitate is the immunoprecipitation complex, which is further detected by Western Blot.

### 2.6 GST-pull down assay

The GST-TGFBR3 fusion protein was cloned into the expression vector pGEX-4T-1 (HG-P003243, Changsha, China) and purified from BL21 (DE3) bacterial cultures. The purified fusion protein was subsequently immobilized onto glutathione–Sepharose beads (C4-0531-01-03, Seplife, Xi’an, China). GST protein expressed from the empty vector was similarly purified and used as a negative control. A total of 10 μg of purified FTAP2-His protein (bs-105227P, Bioss, Beijing, China) was added to the beads pre-immobilized with either GST-TGFBR3 or GST, and the mixtures were incubated overnight at 4 °C on a rotating shaker. After incubation, the beads were washed four to five times with wash buffer to remove non-specifically bound proteins. Subsequently, 40 μL of 1× SDS sample loading buffer was added, and the samples were boiled at 95 °C for 5 minutes. Following centrifugation, the supernatants were collected for further analysis.

### 2.7 CCK-8 assay

Cell proliferation was evaluated using the CCK-8 method. Collect cells in logarithmic growth phase, adjust cell density, and inoculate 8 × 10 ³ cells per well into a 96 well plate. After cell adhesion, different concentrations of FATP2 inhibitors (Lipofermata, MedChemExpress, purity: 99.53%)(0, 10, 20, 30, 50, 100, 200, and 500 μM) were added. After 24 hours of cultivation, add 10 μ L of CCK-8 reagent to each well and continue incubating for 1.5 hours. Subsequently, the absorbance value at a wavelength of 450 nm was measured using an enzyme-linked immunosorbent assay (Thermo Scientific, China).

### 2.8 Wound healing assay and invasion experiment

The cell migration ability was evaluated through Wound healing assay: Wound healing assays were made when the cell fusion reached 95% -100%, and a vertical incision was made using a pipette with a volume of 20 microliters at the center of each well. Subsequently, the board was cleaned three times to remove scraped cells. Replace with 2% serum culture medium, take photos at 0, 24, and 48 hours, and calculate migration rate using Image J. The cell invasion ability was evaluated by Transwell assay: matrix gel (1:8 dilution) was coated in a small chamber, and 10% FBS was added as a chemotactic factor in the lower chamber. Cell suspension of 1 × 10^5^ -2 × 10^5^ cells/mL was inoculated in the upper chamber, incubated for 24 hours, fixed, stained with crystal violet, and counted under a microscope. The experiment was independently repeated three times.

### 2.9 Clinical sample collection

This study protocol was approved by the Ethics Committee of Hunan Brain Hospital (The Second People’s Hospital of Hunan Province) (Approval No. 2025K055). Written informed consent was obtained from all participants prior to enrollment.

Patients with newly diagnosed, treatment-naïve lung cancer admitted to Hunan Brain Hospital between January 2024 and December 2025 were enrolled. All eligible patients during the study period were consecutively included. The diagnosis of lung cancer was pathologically confirmed according to the WHO classification of lung tumors. Healthy controls and pneumonia controls were recruited from the same hospital during the same period (from the Health Examination Center and the Respiratory Medicine Ward, respectively).

Inclusion criteria for the lung cancer group were (1): newly diagnosed primary lung cancer, pathologically confirmed (2); no prior antitumor therapy before blood collection (3); age ≥ 18 years; and (4) complete clinical data available. Exclusion criteria were (1): concurrent malignancies (2); severe hepatic or renal dysfunction (3); autoimmune diseases or active infections; and (4) pregnancy or lactation. The exclusion criteria for healthy controls and pneumonia controls were the same as items (2) through (4) for the lung cancer group, with the additional requirement that healthy controls had no history of pulmonary disease.

Peripheral venous blood samples were collected from all participants in the morning after an overnight fast (≥ 8 hours) and processed immediately upon collection. Whole blood was centrifuged at 3000 rpm for 10 minutes at 4 °C to separate serum. The serum was then aliquoted into 1.5 mL EP tubes, each labeled with a unique sample identifier, and stored at −80 °C until further analysis. All samples were protected from repeated freeze-thaw cycles.

Demographic and clinical characteristics, including age, sex, body mass index (BMI), smoking status, comorbidities (hypertension, diabetes, cardiovascular disease), use of lipid-lowering medications, and tumor stage in lung cancer patients, were collected from the electronic medical record system. All data were obtained at the time of initial diagnosis, prior to any therapeutic intervention.

### 2.10 ELISA experiment

Serum and cell culture supernatant levels of sTGFBR3 and FATP2 were measured using commercial ELISA kits according to the manufacturers’ instructions (sTGFBR3: Huamei Biotechnology, Cat. No. CSB-EL023453HU; FATP2: Wuhan Kelu Biological, Cat. No. ELK0461). All samples were assayed in duplicate.

The TGFBR3 ELISA kit had a detection range of 0.156–10 ng/mL, a sensitivity of 0.039 ng/mL, and intra- and inter-assay coefficients of variation (CVs) of < 8% and < 10%, respectively. This kit specifically detects human TGFBR3 with no significant cross-reactivity with other related proteins.

For the FATP2 ELISA kit, the detection range was 0.16–10 ng/mL, with a sensitivity of 0.068 ng/mL. Intra-assay precision was assessed by testing three samples of known concentrations in 20 replicates on a single plate (CV < 8%), and inter-assay precision was assessed by testing the same three samples in 40 replicates across three different plates (CV < 10%).

Values below the limit of detection were recorded as LOD/2 for statistical analysis; samples above the upper limit of quantification were diluted and re-assayed, with results corrected by the dilution factor.

### 2.11 Protein immunoblotting experiment

Extract total protein using RIPA lysis buffer. The protein extract was then separated by SDS-PAGE gel electrophoresis. The separated protein is transferred to a PVDF membrane. Subsequently, the specific antibody was incubated with PVDF membrane at 4 °C. Proteins are detected using enhanced chemiluminescence reagents. The main antibodies used in this study include anti-TGFBR3 (DF8749,1:1000), anti-FATP2 (14048-1-AP,1:2000), anti-ACSL1 (13989-1-AP,1:1000), anti-ACC1 (21923-1-AP,1:5000), and anti-SCD1 (28678-1-AP,1:1000). Anti-CD36(18836-1-AP,1:500) and anti-GAPDH(60004-1-Ig,1:25000).

### 2.12 Statistical analysis

All statistical analyses were conducted using SPSS 20.0 and GraphPad Prism 7, with one-way analysis of variance and non-parametric tests. Data from at least three independent experiments are presented in standard deviation (SD) format. The difference is considered significant, *P* < 0.05(*P < 0.05, **P < 0.01, ***P < 0.001).

## 3 Results

### 3.1 Dyslipidemia is present at the time of initial diagnosis of lung cancer

**Figure 1:**
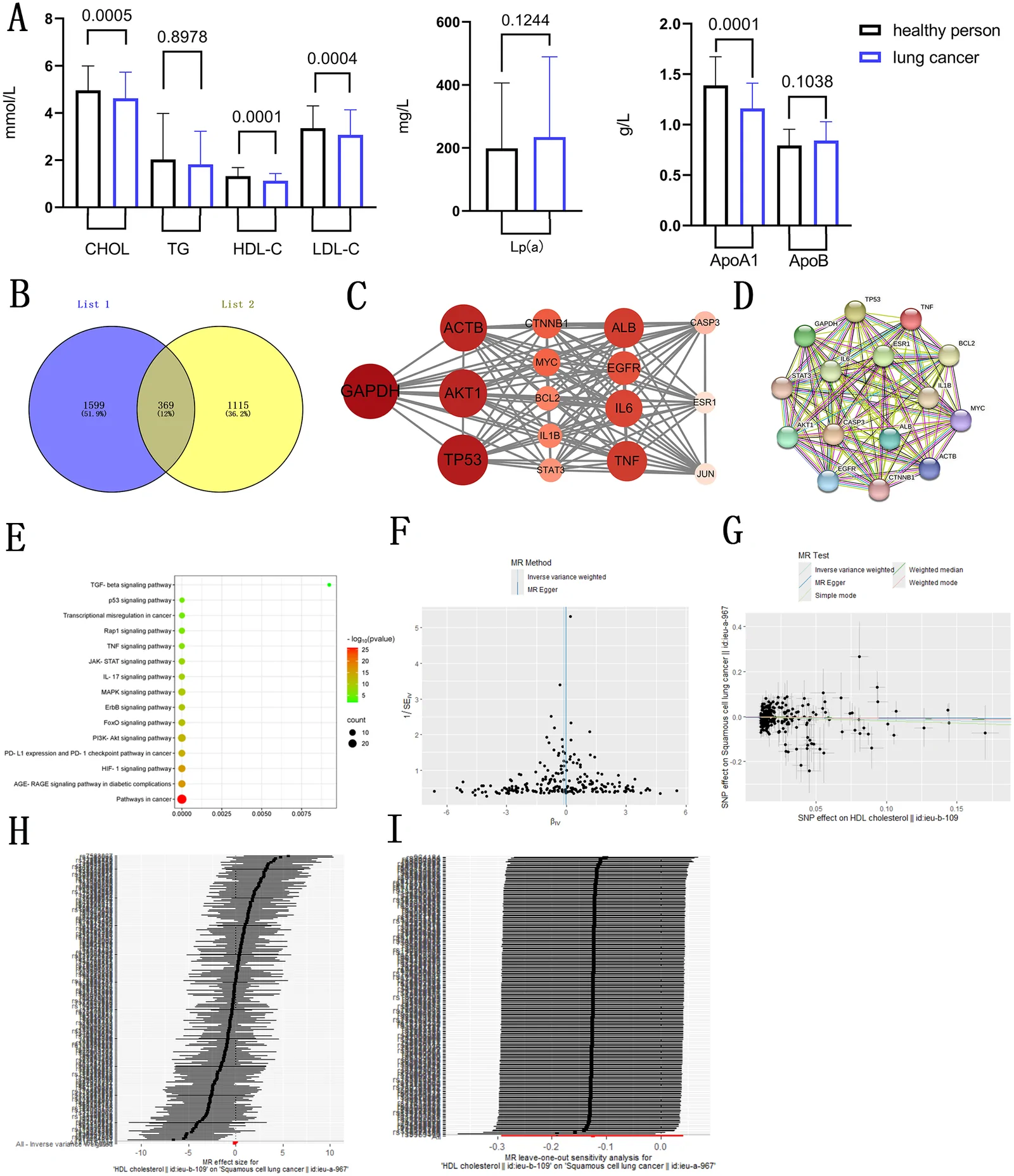
Lipid metabolism and lung cancer. **(A)** Comparison of seven serum lipid parameters between healthy controls and patients with newly diagnosed lung cancer. Levels of CHOL, HDL-C, LDL-C, and ApoA1 were significantly decreased in lung cancer patients (P < 0.05). **(B)** Venn diagram of shared targets between lipid metabolism dysregulation and lung cancer. **(C)** Network analysis of core targets. **(D)** PPI network of core targets. **(E)** Bubble plot of top 15 KEGG pathway enrichment analyses of core targets. **(F)** Funnel plot assessing potential publication bias; symmetrical distribution suggests no significant bias. **(G)** Scatter plot showing the association between each SNP and HDL-C levels (x-axis) and its effect on lung squamous cell carcinoma risk (y-axis). Slopes represent causal estimates from various MR methods (IVW, weighted median, MR-Egger, weighted mode, and simple mode). **(H)** Forest plot presenting the individual and combined causal effect estimates of each SNP on the association between HDL-C and lung squamous cell carcinoma. Horizontal lines represent 95% confidence intervals; the red segment indicates the pooled estimate using the IVW method. **(I)** Leave-one-out analysis forest plot showing the combined effect estimates after sequentially excluding each SNP, used to assess the influence of individual SNPs on the overall causal estimate.

A total of 30 healthy controls, 30 patients with pneumonia, and 170 patients with newly diagnosed lung cancer were enrolled in this study. Among the lung cancer patients, 26 received at least four cycles of chemotherapy (21 days per cycle), while the remaining patients discontinued chemotherapy due to personal reasons. As shown in (**Figure 1A**), patients with newly diagnosed lung cancer exhibited dyslipidemia, characterized by decreased levels of CHOL, HDL-C, LDL-C, and ApoA1.

### 3.2 The correlation between lung cancer and lipid metabolism

By database screening, 1,484 targets related to lipid metabolism disorders and 1,970 lung cancer-related targets were obtained. The intersection of the two yielded 369 common targets (**Table 2**), including multiple members of the TGF-β family and the key fatty acid metabolism molecule FASN. Based on the common targets, a protein-protein interaction network was constructed, and topological analysis identified 15 core targets. KEGG pathway enrichment analysis showed that the relevant pathways were mainly enriched in the TGF-β signaling pathway (**Figures 1B–E**).

**Table 2:** Common targets of lipid metabolism and lung cancer.

Gene symbol						
MMP9	STAT3	TGFB1	GAPDH	CTNNB1	JUN	CCND1
CASP3	BCL2	IGF1	AKT1	EGFR	IL6	MYC
ACTB	SRC	TLR4	KRAS	TNF	MTOR	HIF1A
TP53	NFKB1	GSK3B	IFNG	CDH1	ESR1	PPARG
ALB	PTEN	EGF	IL1B	SIRT1	FN1	FOXO1
ERBB2	HPRT1	ODC1	ATP2A2	STAR	TNFSF11	FLNC
PIK3CD	MMP1	ID2	TFAP2A	TGFBR1	HDAC4	CAPN3
FOS	TGFBR2	G6PD	APP	NGF	MET	IL6R
FASN	CYCS	F3	LOX	RGS4	FOXP1	RELN
PARP1	NOS2	EDNRB	GGT1	RRM2B	PTCH1	CALCA
NFKBIA	GSK3B	PIK3CA	SCD	GRN	SDHA	PRKN
RB1	MAP2K1	ABCA1	RARB	FGFR2	DPP4	PMS2
RUNX1	PIK3CA	XDH	TGFB2	MAOA	TCF4	SFTPB
GSTM1	ATM	TNFRSF1A	EZH2	DNMT3B	CSF1R	……

The summary statistical data of exposure factor HDL-C is sourced from the IEU OpenGWAS database (GWAS ID: ieu-b-109), which is based on the European population. The outcome data was sourced from the GWAS summary data of lung squamous cell carcinoma (GWAS ID: ieu-a-966), also from the European population. Mendelian randomization analysis showed (**Figures 1F–I**) that genetically predicted HDL-C levels were significantly negatively correlated with the risk of squamous cell lung cancer based on 227 SNPs selected as instrumental variables. The weighted median method, which provides consistent estimates even when up to 50% of the instruments are invalid, yielded a significant causal estimate (OR = 0.5607, 95% CI: 0.3733–0.8423, P = 0.0056). Consistent directional effects were observed across other MR methods: IVW random-effects model showed a protective but non-significant trend (OR = 0.88, 95% CI: 0.75–1.04, P = 0.142), MR-Egger (OR = 0.98, 95% CI: 0.76–1.27, P = 0.890), simple mode (OR = 0.83, 95% CI: 0.48–1.43, P = 0.494), and weighted mode (OR = 0.93, 95% CI: 0.71–1.22, P = 0.611) all showed consistent protective direction. The MR-Egger intercept test did not find significant horizontal pleiotropy (intercept = -0.0042, SE = 0.0039, P = 0.281), suggesting that the genetic instruments are unlikely to affect lung cancer risk through pathways independent of HDL-C. Cochran’s Q test revealed substantial heterogeneity among the SNPs (IVW: Q = 283.9, P = 0.005; MR-Egger: Q = 282.5, P = 0.006), which justified the use of a random-effects model for the IVW method and strengthened our reliance on the weighted median estimate as the primary finding. Leave-one-out analysis further confirmed the stability of the findings, as no single SNP disproportionately drove the overall causal estimate.

### 3.3 Interaction between FATP2 and TGFBR3

**Figure 2:**
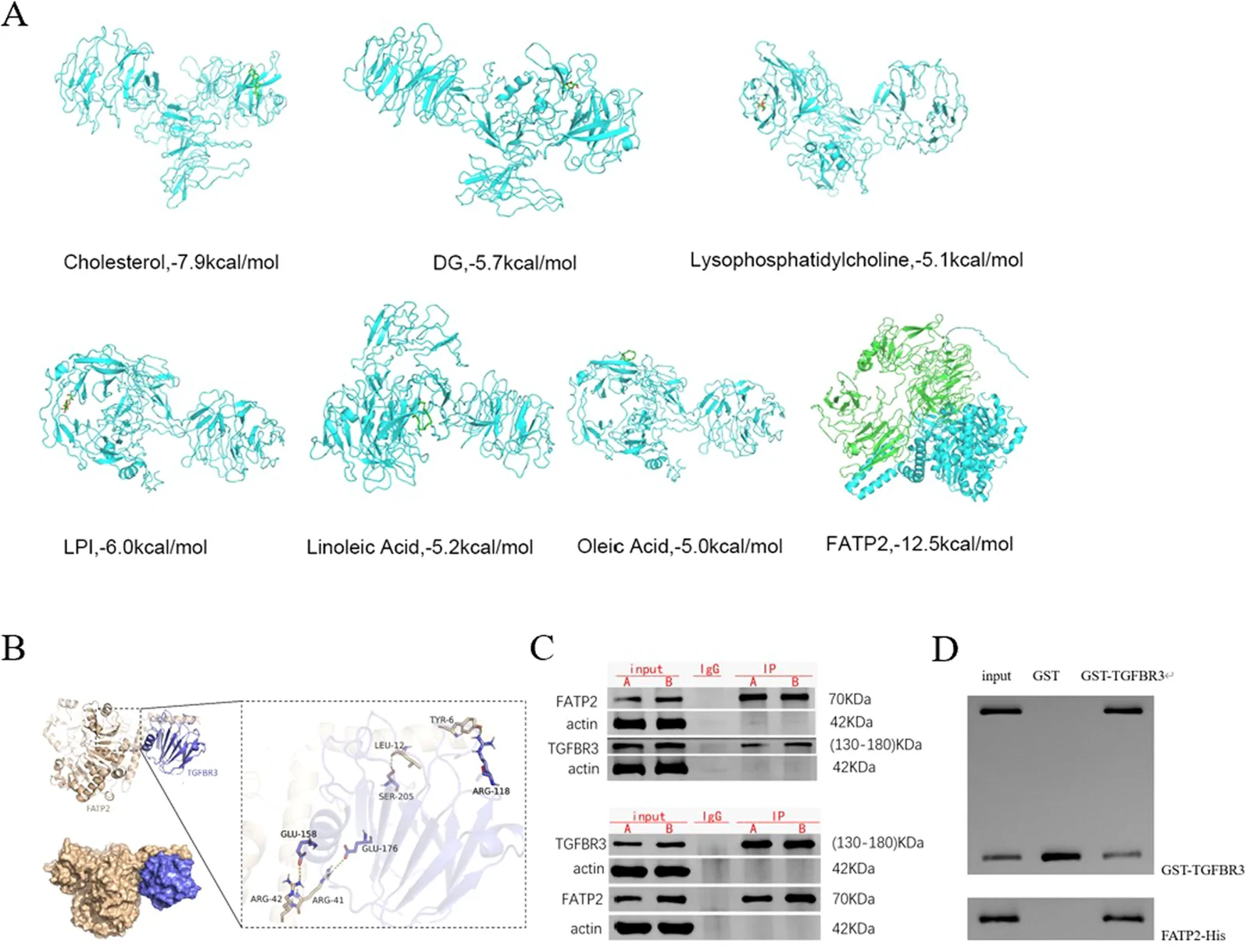
Validation of TGFBR3-FATP2 interaction via molecular docking and co-immunoprecipitation. **(A)** Molecular docking results and binding energies of lipid metabolism-related proteins with TGFBR3. Binding energies are expressed in kcal/mol; lower values indicate more stable binding. **(B)** Binding mode of the FATP2-TGFBR3 complex. FATP2 is shown in wheat, TGFBR3 in blue. Yellow dashed lines indicate hydrogen bonds. **(C)** Co-IP analysis of TGFBR3 and FATP2 interaction in A549 and NCI-H292 cells. Input: cell lysates showing basal expression levels of FATP2 and TGFBR3. IgG: negative control with no non-specific bands. Actin (42 kDa) served as a loading control. Lane 1: A549 cells; Lane 2: NCI-H292 cells. **(D)** GST-TGFBR3 or GST empty vector proteins were incubated with FATP2-His and subjected to pull-down assays. GST antibody detection confirmed successful enrichment of GST-TGFBR3. His antibody detection revealed that the FATP2-His band was detected in the GST-TGFBR3 group, but not in the GST empty vector group, indicating a direct interaction between TGFBR3 and FATP2.

Through network pharmacology, Mendelian randomization analysis, and literature research, potential protein targets related to TGFBR3 in the lipid metabolism pathway were screened, and their interactions were validated by molecular docking. All complexes have negative binding energies, with TGFBR3 binding to FATP2 at -12.5 kcal/mol. The predicted complex structure by ZDOCK shows the formation of multiple hydrogen bonds between FATP2 and TGFBR3, including ARG-42, ARG-41, LEU-12 of FATP2, GLU-158, GLU-176, SER-205, and ARG-118 of TYR-6 and TGFBR3 (**Figures 2A, B**).

To validate the biological plausibility of the TGFBR3-FATP2 complex predicted by molecular docking, we further analyzed the structural topology of both proteins.

The TGFBR3 structure used in this study was derived from the PDB database (ID: 7LBG), which encompasses the major extracellular domain of TGFBR3 (aa 1-781). The FATP2 structure was obtained from the AlphaFold database. According to UniProt annotation (O14975), the N-terminal transmembrane region of FATP2 is located at aa 5-27, followed by a cytoplasmic domain spanning aa 28-106.

Based on the above topological localization, we assessed the accessibility of the predicted interface residues. Our molecular docking results revealed that FATP2 ARG-41 and ARG-42 are situated within the cytoplasmic domain, while TGFBR3 GLU-158 and GLU-176 are located in the extracellular juxtamembrane region. These two pairs of residues form a charge-complementary salt bridge/hydrogen bond network, indicating that the interaction interface between the two proteins is positioned in close proximity to the cell membrane surface—where the juxtamembrane extracellular region of TGFBR3 converges with the cytoplasmic domain of FATP2.

This spatial configuration satisfies the topological orientation constraints of membrane proteins, and all participating residues are exposed to hydrophilic environments, ensuring their biological accessibility. This analysis provides structural rationale for the direct interaction between TGFBR3 and FATP2 at the cell membrane.

Co-ip further confirms that FAYP2 and TGBFR3 can capture each other (**Figure 2C**), which is further supported by the GST pull down assay (**Figure 2D**).

### 3.4 Constructing TGFBR3 lentivirus transfected cell lines

**Figure 3:**
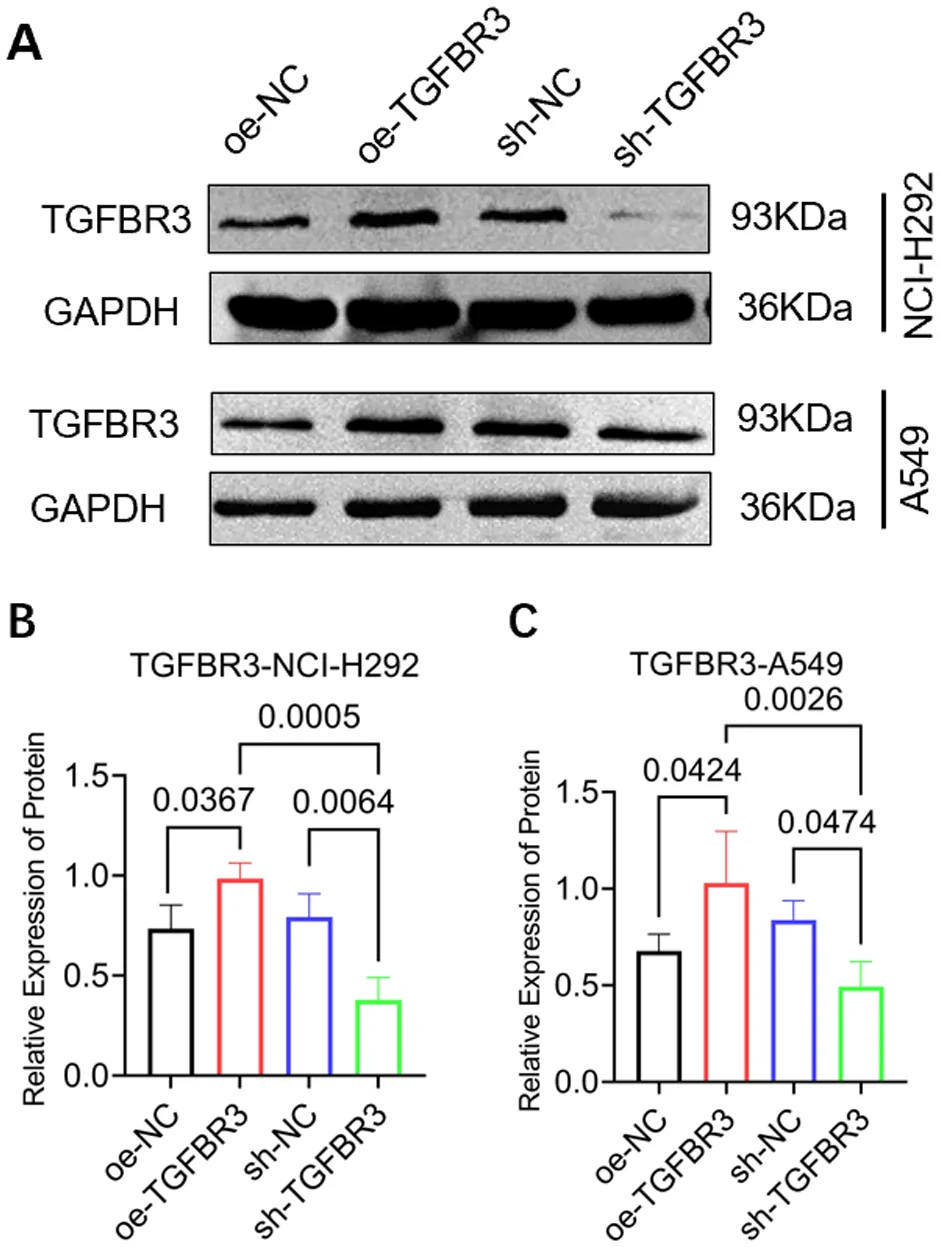
Detection of TGFBR3 expression after lentiviral transduction by Western blotting. **(A)** Western blot analysis of TGFBR3 protein expression after viral transduction. **(B, C)** Validation of stable transfectants in NCI-H292 and A549 cells. TGFBR3 expression was significantly higher in oe-TGFBR3 cells compared to the oe-NC group, and significantly lower in the sh-TGFBR3 group compared to the sh-NC group (P < 0.05).

Using lentiviral vectors (oe NC, oe TGFBR3, sh NC, sh TGFBR3) to transfect A549 and NCI-H292 cells. The optimal multiplicity of infection (MOI) for A549 cells is 10, while that for NCI-H292 cells is 50. After 72 hours of transfection, Use a culture medium containing Purmycin (2ug/ul) for screening. Western blot analysis showed that the TGFBR3 protein expression level in the oe-TGFBR3 group was significantly higher than that in the control group (*P* = 0.0367), while the sh-TGFBR3 group was significantly lower than the control group (*P* = 0.0064, **Figure 3**).

### 3.5 TGFBR3-FATP2 interaction correlates with changes in lung cancer cell migration and invasion

**Figure 4:**
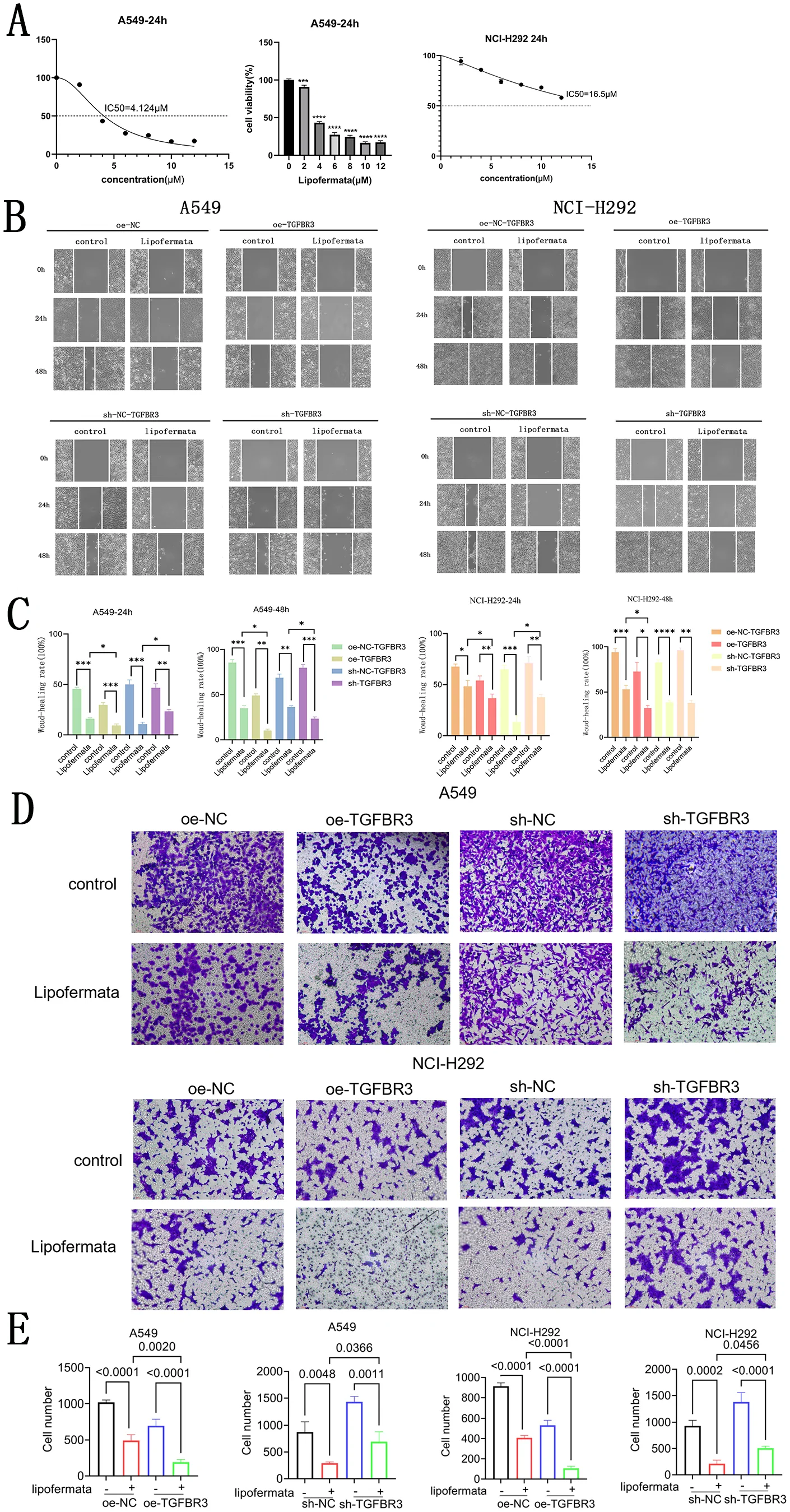
TGFBR3 regulates FATP2 to inhibit lung cancer cell proliferation, migration, and invasion. **(A)** Lipofermata inhibits lung cancer cell proliferation. **(B)** TGFBR3 targets FATP2 to inhibit cell migration. **(C)** Quantitative analysis of cell migration rates from wound healing assay. Lipofermata treatment significantly suppressed migration in A549 and NCI-H292 cells. TGFBR3 overexpression further enhanced this inhibition, while TGFBR3 knockdown partially restored migration ability (P < 0.05). **(D)** TGFBR3 targets FATP2 to inhibit cell invasion. **(E)** Quantitative analysis of cell invasion ability from invasion assay. Lipofermata treatment markedly reduced invasion in A549 and NCI-H292 cells. TGFBR3 overexpression potentiated the inhibitory effect (P < 0.005), whereas TGFBR3 knockdown partially rescued invasion ability (P < 0.05).

Lipofermata treatment was associated with reduced proliferation activity of A549 and NCI-H292 cells, with half maximal inhibitory concentrations (IC _50_) of 4.124 μ M and 15.4 μ M, respectively (*P* < 0.05, **Figure 4A**). Subsequent experiments were performed at the respective IC _50_ concentrations.

Wound healing assay test and Transwell invasion assays revealed that Lipofermata treatment accompanied decreases in cell migration and invasion compared with the control group (*P* < 0.05, **Figures 4B–E**). TGFBR3 overexpression was associated with decreased migration and invasion, whereas TGFBR3 knockdown was associated with relatively increased migration and invasion compared with the overexpression group (*P* < 0.05).

### 3.6 TGFBR3 affects the expression of FATP2-ACSL1 axis related proteins

**Figure 5:**
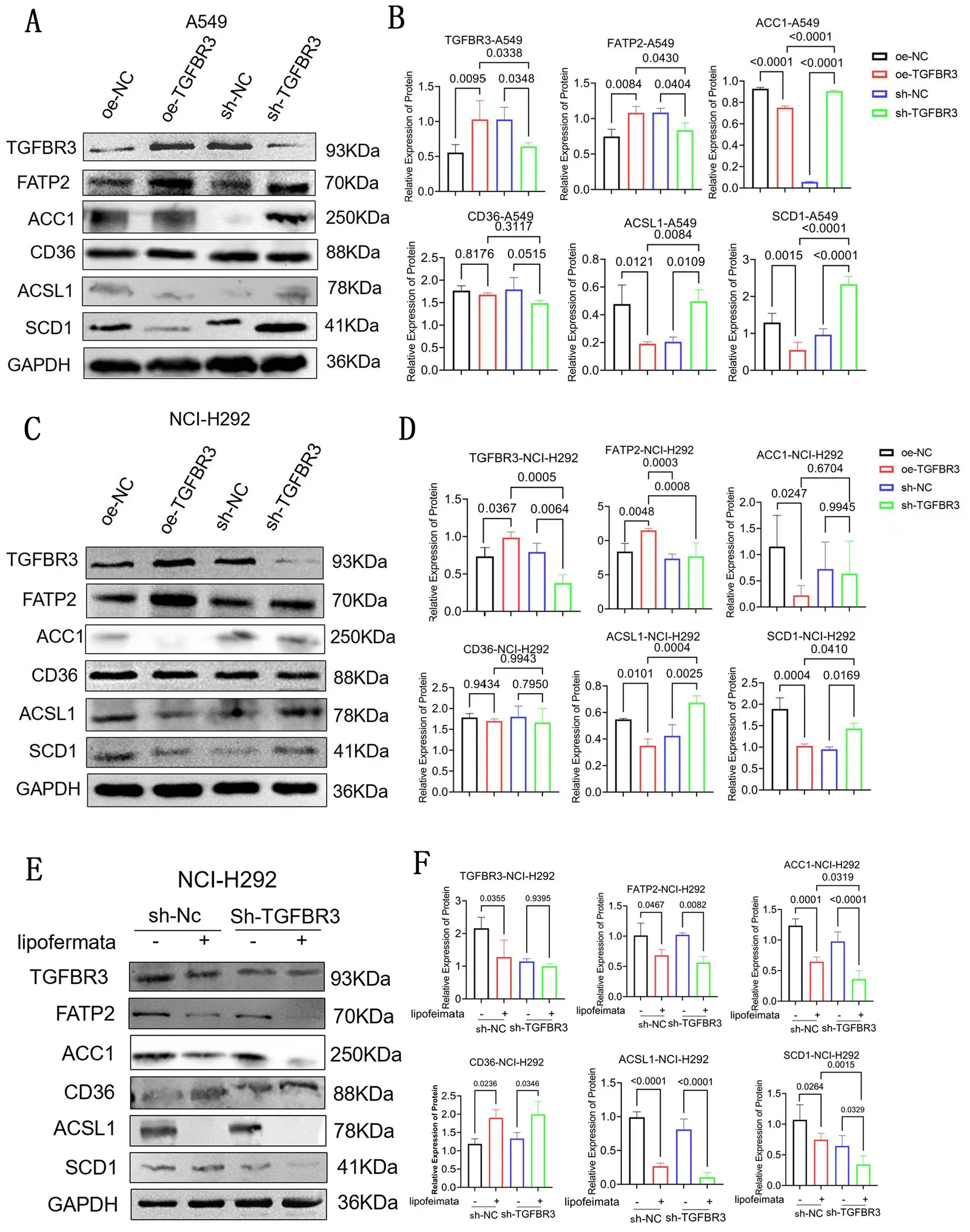
Effects of TGFBR3 on proteins of the FATP2-ACSL1 axis. **(A)** Western blot analysis of related proteins in NCI-H292 cells. **(B)** TGFBR3 modulates the expression of FATP2-ACSL1 axis-associated proteins in NCI-H292 cells. Overexpression of TGFBR3 decreased the protein levels of ACSL1, ACC1, and SCD1 (P < 0.05), while TGFBR3 knockdown restored their expression (P < 0.05). **(C)** Western blot analysis of related proteins in A549 cells. **(D)** TGFBR3 modulates the expression of FATP2-ACSL1 axis-associated proteins in A549 cells. Overexpression of TGFBR3 decreased the protein levels of ACSL1, ACC1, and SCD1 (P < 0.05), while TGFBR3 knockdown restored their expression (P < 0.05). **(E)** Western blot analysis of related proteins in NCI-H292 cells after Lipofermata treatment. **(F)** Lipofermata modulates the expression of FATP2-ACSL1 axis-associated proteins in NCI-H292 cells. Following Lipofermata intervention, the expression of ACC1, TGFBR3, ACSL1, FATP2, and SCD1 decreased (P < 0.05), whereas CD36 expression increased.

Western blot analysis showed in A549 cells and NCI-H292 cell lines (**Figure 5**). There was no difference in the expression of CD36, whether it was overexpression or interference, while the expression of FATP2 showed significant changes. After overexpression of TGFBR3, the expression levels of ACSL1, ACC1, and SCD1 proteins significantly decreased (*P* < 0.05), while interference with TGFBR3 restored the expression of the aforementioned proteins (*P* < 0.05). To further clarify whether TGFBR3 acts through the FATP2-ACSL1 axis, the FATP2 inhibitor Lipofermata was used for intervention. The experiment was divided into four groups: sh-NC-TGFBR3, sh-TGFBR3, sh-NC-TGFBR3+Lipofengmata, and sh-TGFBR3+Lipofergmata. Western blot results showed that after treatment with Lipofermata, the expression of FATP2, TGFBR3, ACC1, ACSL1, and SCD1 decreased (*P* < 0.05), while CD36 expression was upregulated. Under the background of sh-TGFBR3 combined with Lipofermata treatment, the expression of ACC1 and SCD1 further decreased (*P* < 0.05, **Figure 5**).

### 3.7 Clinical level validation of the correlation and clinical value of soluble TGFBR3-FATP2

**Figure 6:**
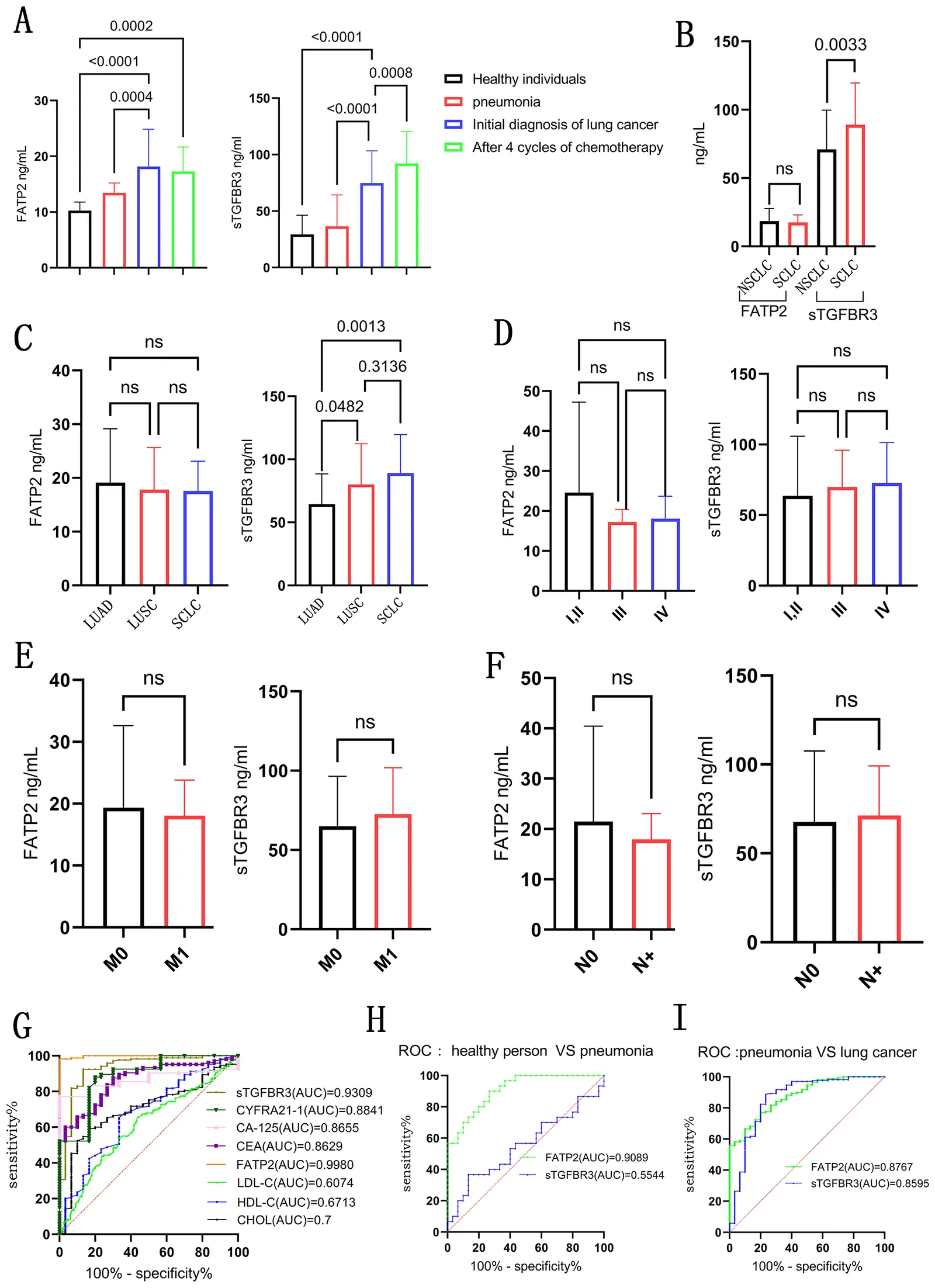
Serum levels of sTGFBR3 and FATP2 and their diagnostic value for lung cancer. **(A)** Serum sTGFBR3 and FATP2 levels in healthy controls, pneumonia patients, and lung cancer patients. sTGFBR3 was significantly elevated in newly diagnosed lung cancer patients and remained elevated after four cycles of chemotherapy. **(B)** Differences in serum levels of sTGFBR3 and FATP2 between non-small cell lung cancer and small cell lung cancer. **(C)** Differences in serum sTGFBR3 and FATP2 levels among different pathological types of lung cancer. **(D)** Differences in serum sTGFBR3 and FATP2 levels among patients with different clinical stages. **(E)** Differences in serum levels of sTGFBR3 and FATP2 between distant metastatic lung cancer and non-metastatic lung cancer. **(F)** Differences in serum levels of sTGFBR3 and FATP2 between lung cancer patients with and without lymph node metastasis. **(G)** ROC curves. **(H)** ROC curve distinguishing healthy controls from pneumonia patients. **(I)** ROC curve distinguishing pneumonia patients from lung cancer patients. (ns indicates no statistical significance).

A total of 30 healthy controls, 30 patients with pneumonia, and 170 patients with newly diagnosed lung cancer were enrolled, with no differences in baseline characteristics among the three groups (**Table 3**). Among the 170 patients with newly diagnosed lung cancer, 23 received at least 4 cycles (21 days/cycle) of chemotherapy, the remaining patients discontinued chemotherapy for personal reasons. Compared with healthy controls and patients with pneumonia, serum levels of sTGFBR3 and FATP2 were significantly increased in patients with newly diagnosed lung cancer. In an exploratory analysis, serum sTGFBR3 levels in the 23 patients after 4 cycles of chemotherapy were higher than those in the overall newly diagnosed lung cancer cohort (n=170) at baseline, but remained above the levels of healthy controls (**Figure 6A**).

**Table 3:** Comparison of baseline data among healthy individuals, pneumonia patients, and lung cancer patients.

	Healthy person (n=30)	Pneumonia (n=30)	Lung cancer (n=170)	*P*
Age (years)				0.3621
Mean ± standard deviation	60.1 ± 14.2	63.0 ± 11.1	63.3 ± 11.0	
Median (interquartile range)	60.5(52.0,70.5)	64.5(53.8, 72.0)	66.0(58.0, 72.0)	
Range (minimum, maximum)	(31, 83)	(41, 84)	(31, 96)	
Gender (Example%)				0.75
male	25 (83.3%)	24 (80.0%)	138 (81.2%)	
woman	5 (16.7%)	6 (20.0%)	32 (18.8%)	
smoking				0.088
no	5	10	68	
yes	25	20	102	
Drinking Wine				0.282
No	17	22	122	
yes	13	8	48	
lipid-lowering drugs				0.348
no	30	30	163	
yes	0	0	7	

A total of 170 patients with newly diagnosed lung cancer were enrolled, including 143 NSCLC cases (81 LUAD, 59 LUSC, and 3 with incomplete clinical data), 26 SCLC cases, and 1 unclassified case. Serum sTGFBR3 levels differed among histological types: both LUSC and SCLC patients had significantly higher levels than LUAD patients (*P* < 0.05), while no significant difference was observed between LUSC and SCLC (*P* > 0.05) (**Figure 6C**). No significant differences in serum FATP2 levels were found among different histological types. Among the 143 NSCLC patients, 18 were stage I+II, 50 were stage III, 73 were stage IV, and 2 were not staged. No significant differences in serum sTGFBR3 or FATP2 levels were observed among different clinical stages (**Figure 6D**, all *P* > 0.05).

When comparing NSCLC with SCLC, serum sTGFBR3 levels in the SCLC group were significantly higher than those in the NSCLC group (*P* < 0.05). In the NSCLC subgroup, although patients with lymph node metastasis (N+) or distant metastasis (M1) had higher median serum levels of sTGFBR3 and FATP2 than those without metastasis (N0/M0), none of these differences reached statistical significance (**Figure 6**; **Table 4**).

**Table 4:** Serum sTGFBR3 and FATP2 levels in NSCLC patients: comparison between metastatic and non-metastatic groups.

	M0 (n=53)	M1 (n=69)	N0 (n=25)	N+ (n=106)
sTGFBR3	64.41 (49, 80.70)	67.15 (49.94, 88.81)	57.35 (42.36, 90.95)	68.04 (52.9, 86.2)
FATP2	16.03 (15.28, 18.06)	16.66 (15.41, 18.7)	15.94 (15.2, 17.38)	16.51 (15.38, 19.17)

ROC curve analysis was performed to explore the diagnostic value of each marker for lung cancer (**Figure 6G**). In this cohort, the area under the curve (AUC) for sTGFBR3 was 0.9, for FATP2 was 0.9978, for conventional tumor markers (such as CEA, CYFRA21-1, and CA-125) ranged from 0.8629 to 0.8841, and for the five lipid parameters ranged from 0.6206 to 0.7. With a cut-off value of 42 ng/mL for sTGFBR3, the sensitivity was 90.0%, specificity was 90.0%, and the Youden index was 0.8. With a cut-off value of 13.15 ng/mL for FATP2, the sensitivity was 98%, specificity was 100%, and the Youden index was 0.98 (**Table 5**). Preliminarily, sTGFBR3 and FATP2 appeared to show higher diagnostic efficacy than the other markers in this cohort, although these findings require validation in independent cohorts. The ability of these two markers to discriminate among the healthy control, pneumonia, and lung cancer groups was further evaluated (**Figures 6H, I**). For distinguishing healthy controls from pneumonia patients, the AUCs of sTGFBR3 and FATP2 were 0.5544 and 0.9089, respectively; for distinguishing pneumonia from lung cancer, the AUCs were 0.8595 and 0.8767, respectively.

**Table 5:** Optimal cutoff and diagnostic efficiency indicators for eight proteins.

Indicator	Comparison	N(case/control)	AUC(95% CI)	Optimal cutoff	Sensitivity (%, 95 %CI)	Specificity (%, 95 %CI)	Positive likelihood ratio	Negative likelihood ratio	Youden index
sTGFBR3	lung cancer vs. Healthy	170/30	0.90(0.90-1.00)	42.00 ng/mL	90.00 (80.00-100.00)	90.00(70.00-100.00)	9.00	0.11	0.80
FATP2	lung cancer vs. Healthy	170/30	0.99(0.99-1.00)	13.15 ng/mL	98.00(94.29-99.45)	100.00(88.65-100.00)	–	0.02	0.98
CEA	lung cancer vs. Healthy	170/30	0.86(0.80-0.93)	3.06 ng/mL	60.00(50.44-68.86)	96.67(83.33-99.83)	18.00	0.41	0.57
CA-125	lung cancer vs. Healthy	170/30	0.87(0.80-0.93)	9.75 U/mL	83.10(73.70-89.70)	83.30(68.10-92.10)	5.00	0.20	0.66
CYFRA21-1	lung cancer vs. Healthy	170/30	0.88(0.81-0.96)	2.20 ng/mL	88.06(78.17-93.82)	80.00(62.69-90.49)	4.40	0.15	0.68
THOL	lung cancer vs. Healthy	170/30	0.70(0.60-0.80)	4.00 mmol/L	40.00(30.00-50.00)	90.00(80.00-100.00)	5.00	0.67	0.30
HDL-C	lung cancer vs. Healthy	170/30	0.70(0.50-0.80)	0.80 mmol/L	20.00(10.00-30.00)	100.00(80.00-100.00)	–	0.80	0.20
LDL-C	lung cancer vs. Healthy	170/30	0.62(0.56-0.69)	3.26 mmol/L	67.80(58.92-75.55)	56.28(49.34-62.99)	1.55	0.57	0.24
sTGFBR3	Healthy vs pneumonia	30/30	0.55(0.40-0.70)	41.46 ng/mL	36.67(21.87-54.49)	86.67(70.32-94.69)	2.75	0.73	0.23
sTGFBR3	lung cancer vs Pneumonia	170/30	0.85(0.77-0.94)	63.46 ng/mL	61.18(53.68-68.18)	90.00(74.38-96.54)	6.12	0.43	0.51
FATP2	Healthy vs pneumonia	30/30	0.90(0.83-0.97)	11.19 ng/mL	90.00(74.38-96.54)	73.33(55.55-85.82)	3.38	0.14	0.63
FATP2	lung cancer vs Pneumonia	170/30	0.87(0.81-0.93)	15.74 ng/mL	66.47(59.08-73.13)	90.00(74.38-96.54)	6.65	0.37	0.56

-, Positive likelihood ratio specificity is 100% and cannot be calculated (denominator is 0).

**Figure 7:**
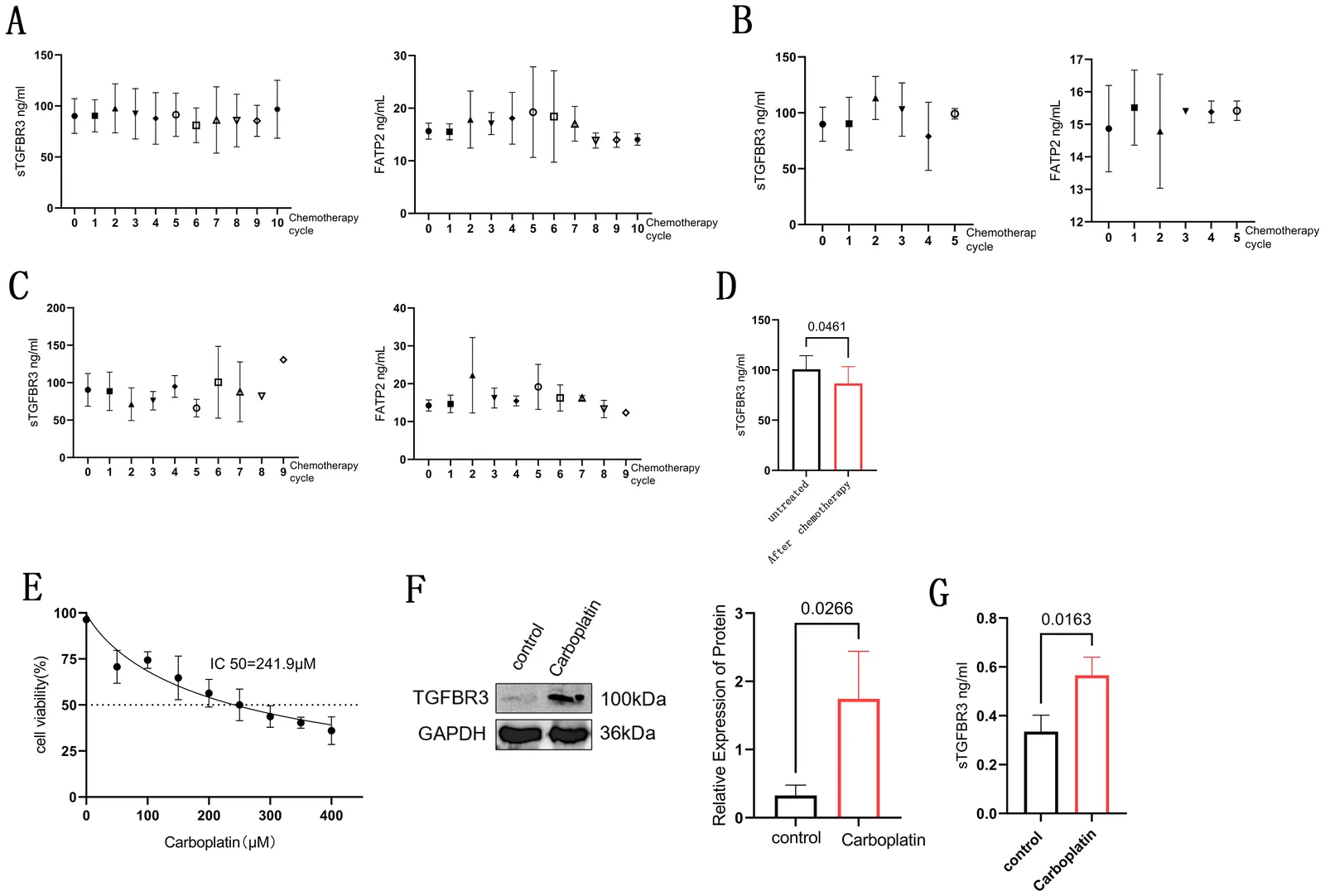
The levels and cellular experimental results of serum sTGFBR3 and FATP2 in lung cancer patients with different therapeutic efficacy groups. **(A)** The levels of sTGFBR3 and FATP2 in lung cancer patients with PR efficacy. **(B)** The levels of sTGFBR3 and FATP2 in lung cancer patients with SD efficacy. **(C)** The levels of sTGFBR3 and FATP2 in lung cancer patients with NA efficacy. **(D)** Differences in sTGFBR3 levels before and after 4 cycles of chemotherapy in 7 lung cancer patients with PR efficacy. **(E)** IC50 of carboplatin on A549 cells for 24 hours. **(F)** Carboplatin affects the expression of TGFBR3 in A549 cells. **(G)** Carboplatin affects the release of sTGFBR3 from A549 cell culture supernatant.

Long-term follow-up showed that at each chemotherapy cycle admission time point, serum sTGFBR3 and FATP2 levels in 23 lung cancer patients exhibited marked individual variability and dynamic changes (**Figures 7A–C**). Most patients showed an inverse dynamic relationship between the two markers: sTGFBR3 tended to decrease while FATP2 tended to increase during the early phase of chemotherapy, with the opposite pattern (sTGFBR3 increase, FATP2 decrease) during the late phase.

Among 15 patients with partial response (PR), 8 showed an overall decreasing or fluctuating decreasing trend in serum sTGFBR3 levels. Further analysis of clinical medication records revealed a temporal association between this decreasing pattern and carboplatin regimen: in 7 patients, sTGFBR3 decreased significantly after carboplatin discontinuation or dose reduction following 4 cycles (*P* < 0.05, **Figure 7D**); in 1 patient, sTGFBR3 decreased after carboplatin discontinuation at the third cycle and increased again after resumption at the fourth cycle. In 2 patients with stable disease (SD), sTGFBR3 initially showed a decreasing trend and then stabilized. In 6 patients with efficacy assessment as NA, the direction of change in sTGFBR3 varied. Notably, marked fluctuations in sTGFBR3 were observed across the entire patient cohort, with a maximum value of 158.9 ng/mL. Patients 9 and 11 showed a pronounced late rebound increase.

To investigate the effect of carboplatin on TGFBR3 expression and soluble release, human lung adenocarcinoma A549 cells were treated with various concentrations of carboplatin (0, 100, 200, 300, 400 µM) for 24 hours. CCK-8 assay results showed that carboplatin at each concentration significantly inhibited the proliferative activity of A549 cells. Using GraphPad Prism for curve fitting, the half-maximal inhibitory concentration (IC_50_) of carboplatin at 24 hours was calculated to be 241.9 µM (**Figure 7E**), and this concentration was used for subsequent experiments. Western blot analysis revealed that after treatment with 241.9 µM carboplatin for 24 hours, TGFBR3 protein expression levels in A549 cells were significantly upregulated compared with the control group (*P* < 0.05, **Figure 7F**). ELISA detection of cell culture supernatants after carboplatin treatment showed that the secretion level of sTGFBR3 in the supernatant of the carboplatin-treated group was significantly increased compared with the control group (*P* < 0.05, **Figure 7G**).

## 4 Discussion

Lung cancer is one of the leading causes of cancer death worldwide, with non-small cell lung cancer accounting for approximately 85% of all lung cancer cases. Tumor metastasis is a core factor leading to poor patient prognosis (26–28). Abnormal activation of lipid metabolism pathways can promote cancer cell proliferation and metastasis (4–6), and cancer cells utilize fatty acid beta oxidation to generate energy to support membrane formation and signal molecule generation (29). Our previous research has shown that TGFBR3 can effectively inhibit the invasion and metastasis ability of lung cancer cells (30), and there is a correlation between TGF - β signaling and lipid metabolism regulation. This study systematically explored the roles of TGFBR3 and lipid metabolism related protein FATP2 in the occurrence, development, and chemotherapy response of lung cancer, from bioinformatics prediction, molecular mechanism validation to clinical translational application.

Through multi database screening and PPI network analysis, this study identified the common target of lipid metabolism and lung cancer, among which FASN, as a key rate limiting enzyme for *de novo* synthesis of fatty acids (31), is a downstream effector molecule of the FATP2-ACSL1 axis. KEGG enrichment analysis revealed that fatty acid metabolism may regulate lung cancer progression through multiple signaling pathways such as PI3K Akt, MAPK, TGF - β, and PD-L1/PD-1. Emerging evidence has further elucidated the interplay between fatty acid metabolism and the tumor immune microenvironment in lung adenocarcinoma. One recent study established a lipid metabolism score (LMS) system and identified MK1775 as a potential sensitizing agent that enhances anti-PD-1 efficacy by modulating lipid crosstalk among tumor cells, tumor-associated macrophages, and CD8^+^ T cells via the PI3K/AKT/mTOR pathway (32). Another study developed a prognostic signature based on 10 fatty-acid-metabolism-related genes, demonstrating its utility in predicting patient prognosis, tumor immune microenvironment infiltration, and immunotherapy response, with MOGAT2 identified as a potential anti-tumor regulator in LUAD (33). These findings collectively highlight the critical role of fatty acid metabolism in both tumor immunity and therapeutic outcomes. Mendelian randomization analysis supports the causal relationship between lipid metabolism and lung cancer, suggesting that HDL-C may be a potential protective factor for lung cancer. Significant heterogeneity was detected between instrumental variables in MR analysis (Cochran’s Q test P = 0.005), indicating that genetic instrumental variables may affect outcomes through mechanisms beyond the exposure of interest. Therefore, our research findings should be interpreted as preliminary correlation evidence supporting the potential protective role of HDL-C in lung squamous cell carcinoma, rather than definitive proof of causality. Heterogeneity also emphasizes the necessity of validating and further studying potential biological mechanisms in independent cohorts. Molecular docking and immunoprecipitation experiments confirmed that TGFBR3 specifically binds to fatty acid transporter FATP2, forming a stable protein complex in lung cancer cells. FATP2, as a key protein for long-chain fatty acid uptake, acts as a “gatekeeper” in fatty acid transport (34). Dysfunction of FATP2 can lead to an increase in free fatty acid uptake, which in turn affects cellular metabolism (35). The FATP2-ACSL1 axis synergistically mediates the uptake, activation, and subsequent metabolism of fatty acids, providing energy support for tumor growth. The physical interaction between TGFBR3 and FATP2 suggests that they may jointly affect the expression of fatty acid metabolism related proteins through interactive regulation, thereby driving malignant progression of lung cancer.

Functionally, TGFBR3 regulates the expression of FATP2 and downstream fatty acid metabolism-related proteins (ACSL1, ACC1, and SCD1), accompanying changes in the migration and invasion of lung cancer cells.FATP2 inhibitor Lipofermata can inhibit lung cancer cell viability, overexpression of TGFBR3 can significantly inhibit cell migration and invasion ability, while interference with TGFBR3 can partially restore these abilities. The regulatory effect of TGFBR3 on fatty acid metabolism-related protein expression is mediated not by the classical CD36-dependent transmembrane transport pathway, but by FATP2. As the rate limiting enzyme in the fatty acid desaturation pathway, the loss of SCD1 expression can lead to the accumulation of saturated fatty acids, which in turn affects lipid homeostasis (36, 37).

Based on the above molecular and cellular evidence, this study further explored the potential translational value of serum sTGFBR3 and FATP2 in a clinical cohort. Newly diagnosed, treatment-naïve lung cancer patients exhibited baseline dyslipidemia, accompanied by elevated serum levels of sTGFBR3 and FATP2, suggesting that both markers may be involved in lipid metabolism remodeling associated with lung cancer. In distinguishing lung cancer from pneumonia patients, sTGFBR3 and FATP2 showed promising discriminatory ability, with higher AUC values compared to conventional lipid parameters and certain traditional tumor markers in this cohort. Simple tumor markers often increase in an inflammatory state, making it difficult to distinguish between inflammation and cancer. STGFBR3 and FATP2 exhibit different expression patterns between pneumonia and lung cancer patients, suggesting that they may be regulated by different mechanisms of tumor microenvironment and inflammatory signaling, and have unique complementary advantages in lung cancer screening or early auxiliary diagnosis. In terms of transfer assessment, the expression value of sTGFBR3 in the non-transfer group was slightly higher than that in the transfer group, but did not reach statistical significance, which may be related to the large difference in sample size between the two groups.

During chemotherapy, sTGFBR3 and FATP2 exhibit reverse temporal changes: in the early stage, sTGFBR3 decreases while FATP2 increases, and in the later stage of chemotherapy, the opposite is true. This reverse pattern suggests that tumor cells may have a temporal response mechanism of early metabolic adaptation and late recovery of anti-cancer signals during chemotherapy. The sustained decline of sTGFBR3 was associated with favorable response to chemotherapy, suggesting that it may serve as a potential candidate biomarker for predicting treatment outcomes. However, given the exploratory nature of this finding, its utility in identifying chemotherapy beneficiaries in the early stages warrants further validation in prospective cohorts. It is worth noting that there is a clear temporal correlation between the dynamic changes of sTGFBR3 and the carboplatin medication regimen: sTGFBR3 significantly decreases after discontinuing or reducing carboplatin, and increases again after resuming medication. This phenomenon suggests that carboplatin may directly induce the release of membrane-bound TGFBR3 into soluble form. *In vitro* experiments showed that carboplatin treatment upregulated TGFBR3 protein expression and increased the levels of its soluble form in the culture supernatant, which is consistent with the clinical observations. The marked fluctuations or late rebound of sTGFBR3 levels observed in some patients may reflect dynamic changes in tumor burden, chemotherapy-induced cellular stress, or early signals of treatment resistance. However, it remains possible that these fluctuations may also arise from nonspecific cellular injury or protein shedding. Therefore, while sTGFBR3 variability shows preliminary association with prognosis and drug resistance, its utility as a monitoring marker warrants further investigation.

In summary, serum sTGFBR3 and FATP2 show preliminary potential in the diagnosis and inflammatory differentiation of lung cancer, as well as in predicting treatment response. A sustained decline in sTGFBR3 levels was associated with chemotherapy benefit, while its rebound or marked fluctuations correlated with the risk of drug resistance. The temporal association with carboplatin therapy provides preliminary clues for monitoring the biological effects of chemotherapy drugs.

Several limitations of this study should be acknowledged. First, the relationship between TGFBR3 and the FATP2-ACSL1 axis is primarily based on protein expression analyses and pharmacological inhibitor assays, without genetic rescue experiments to establish causality. The Lipofermata concentrations used were close to the IC_50_, where approximately half of the cells lost viability, and the observed reductions in migration and invasion may therefore be partially attributable to nonspecific cytotoxicity. Second, our metabolic conclusions are limited to serum lipid profiling and expression changes of metabolism-related proteins, without direct measurements of fatty acid uptake, β-oxidation, or metabolic flux. Third, the carboplatin-associated sTGFBR3 elevation may be partly due to nonspecific cellular injury rather than specific cleavage pathway activation, as we did not perform cell death controls or shedding mechanism assays. Fourth, the chemotherapy follow-up cohort included only 23 patients from a single center, and the findings require validation in larger independent cohorts. Finally, all experiments were conducted *in vitro* and lack *in vivo* animal model validation. Therefore, the current conclusions should be regarded as exploratory findings that warrant further investigation. In our future work, we plan to systematically validate the core findings of this study through genetic rescue experiments, direct measurements of fatty acid metabolic function, and *in vivo* animal models, to further elucidate the precise mechanisms underlying the cooperative role of TGFBR3 and FATP2. We also plan to expand the sample size through multicenter collaboration and validate the diagnostic and monitoring value of FATP2/sTGFBR3 in independent cohorts.

## 5 Conclusions

TGFBR3 interacts with FATP2 and is associated with the regulation of fatty acid metabolism-related proteins, accompanying changes in the proliferation, migration, and invasion of lung cancer cells. The use of FATP2 inhibitor Lipofermata or regulation of TGFBR3 expression can effectively intervene in this process. Clinical serum sample analysis revealed associations between sTGFBR3 and FATP2 levels and lung cancer progression. These findings suggest that sTGFBR3 and FATP2 may represent candidate biomarkers for further evaluation; however, independent validation is required prior to clinical application. Despite some limitations, our findings suggest that targeting TGFBR3 and FATP2-ACSL1 axis-related proteins may offer a potential avenue for further investigation in lung cancer treatment.

## Data Availability

The raw data supporting the conclusions of this article will be made available by the authors, without undue reservation.
