# Supplementary material for: TGFBR3 regulates the expression of FATP2-ACSL1 axis related proteins and accompanies changes in the migration and invasion ability of lung cancer cells
Source: Front Oncol. 2026 Aug 12;16:1899185. doi: 10.3389/fonc.2026.1899185 (PMC13506718; doi:10.3389/fonc.2026.1899185)
Supplement: Supplementary file 6 [file DataSheet6.zip › wb 4/FATP2/FATP2-H292-A549/use/1.pdf]

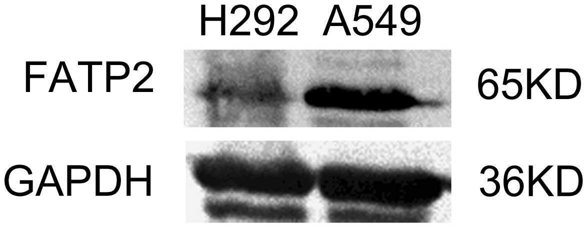
H292
A549
FATP2
65KD
GAPDH
36KD
